# Supplementary material for: Diet Supplementation with Fish-Derived Extracts Suppresses Diabetes and Modulates Intestinal Microbiome in a Murine Model of Diet-Induced Obesity
Source: Mar Drugs. 2021 May 11;19(5):268. doi: 10.3390/md19050268 (PMC8151984; doi:10.3390/md19050268)
Supplement: Supplementary file 1 [file marinedrugs-19-00268-s001.zip › marinedrugs-1201777-supplementary.pdf]

marine drugs

Supplementary Material for

Diet supplementation with fish-derived extracts suppresses diabetes and modulates intestinal microbiome in a murine model of high-fat induced obesity

Konstantinos Axarlis[1,2], Maria G. Daskalaki[1,2], Sofia Michailidou[3], Nikolais Androulaki[1], Antiopi Tsoureki[3], Evangelia Mouchtaropoulou[3], Ourania Kolliniati[1,2], Ioanna Lapi[1,2], Eirini Dermitzaki[1,2], Maria Venihaki[1], Katerina Kousoulaki[4], Anagnostis Argiriou[3,5], Zouhir El Marsni[6,*] and Christos Tsatsanis[2,*]

1 Laboratory of Clinical Chemistry, Medical School, University of Crete, Heraklion 70013, Greece; molgrad392@edu.biology.uoc.gr (KA); m.daskalaki@med.uoc.gr (MGD); nikolandroulaki@gmail.com (NA); raliakolliniatis21@gmail.com (OK); iwanna_lapi@hotmail.com (IL); renaderm@med.uoc.gr (ED); venihaki@med.uoc.gr (MV)
2 Institute of Molecular Biology and Biotechnology, FORTH, 71100 Heraklion, Greece;
3 Institute of Applied Biosciences (INAB), CERTH, Thermi-Thessaloniki GR-57001, Greece; sofia_micha28@certh.gr (SM), adatsoureki@certh.gr (AT); eva.mouchtaropoulou@certh.gr (EM), argiriou@certh.gr (AA)
4 Department of Nutrition and Feed Technology, Nofima AS, 5141 Bergen, Norway; katerina.Kousoulaki@Nofima.no (KK)
5 Department of Food Science and Nutrition, University of the Aegean, Myrina, 81400, Lemnos, Greece;
6 Seagarden AS, 4262 Avaldsnes, Norway;
* Correspondence: tsatsani@med.uoc.gr, Tel.: +30-2810394833 (CT); zouhir.el.marsni@seagarden.no, Tel: +4752859482 (ZEM)

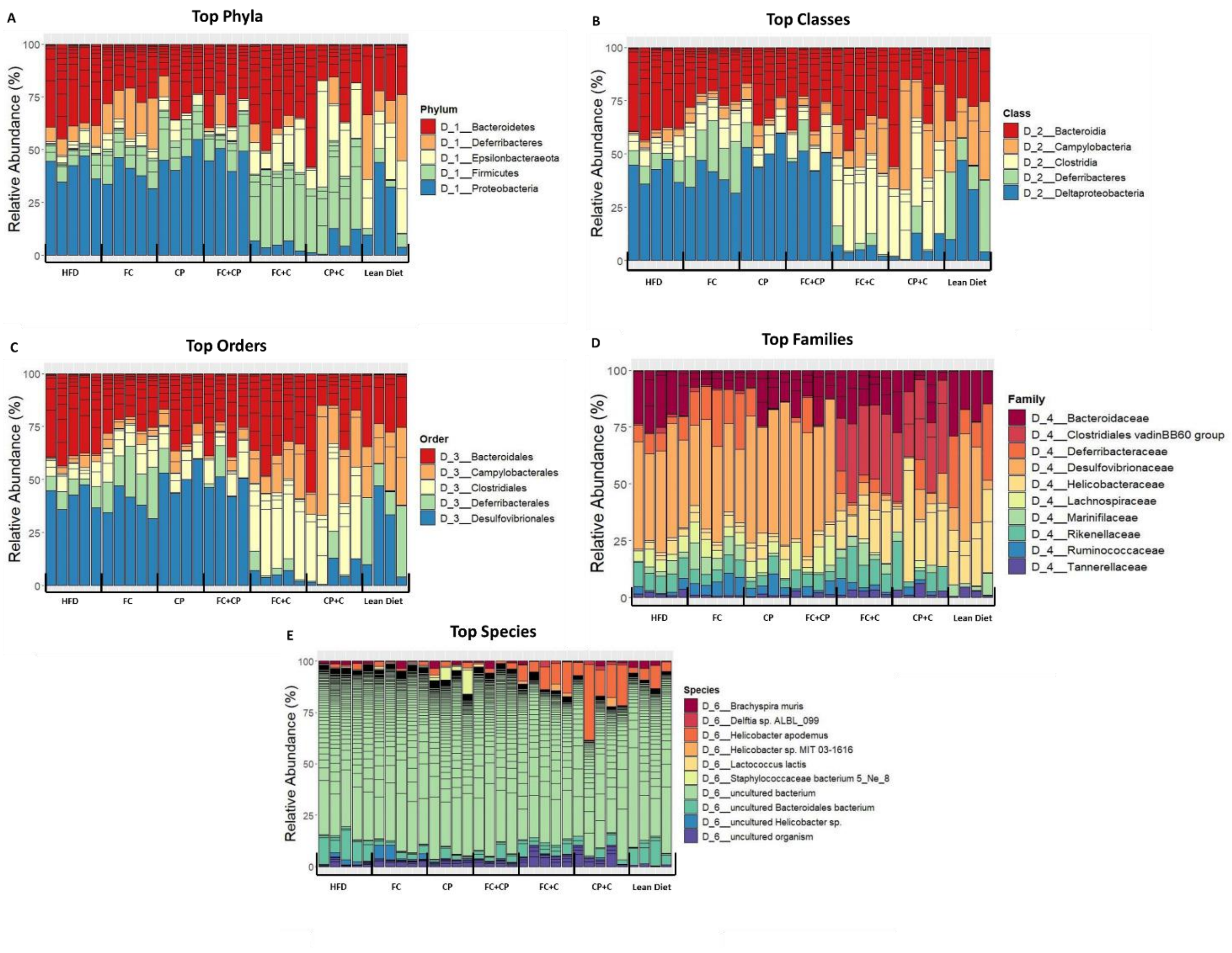

Figure S1. The effect of fish-derived dietary supplements on the intestinal microbiome composition of top A. phyla, B. classes, C. orders, D. families and E. species. Top genera are depicted in the main paper.

Table S1. Nutritional composition of supplemented diets given to each diet group.

**Chemical composition**	**Unit** (per 100g of supplemented food)	**Diet Groups** S (ND)	S (HFD)	FC	CP	FC+CP	FC+C	CP+C
Energy	*Kcal*	272.4	537.8	538.0	534.0	536.0	538.1	536.1
	KJ	1138.8	2253.3	2254.8	2237.5	2246.1	2255.1	2246.5
Carbohydrates	*g*	44.0	24.6	24.6	24.6	24.6	24.6	24.6
Protein	*g*	22.3	35.2	34.8	33.8	34.3	35.0	34.5
Fat	*g*	2.9	33.2	33.4	33.4	33.4	33.3	33.3

Table S2. Summary of top genera found in the intestinal microbiome of high-fat induced obese mice and their role in inflammation and obesity.

Phylum	Genus	Role	Reference
Bacteroidetes	*Bacteroides*	Decreased during IBD, protect against pathogenic microorganism intestinal colonization	[1-3]
	Alistipes	Found in healthy individuals, protection against colitis	[4]
	Odoribacter	Short-chain fatty acid (SCFAs) producers	[5]
Firmicutes	*Lachnospiraceae NK4A136 group*	Butyrate producers, SCFAs	[6,7]
	Ruminiclostridium 9	Butyrate producers, SCFAs	[8]
	Ruminiclostridium	Acetate producers, SCFAs	[9]
Deferribacteres	*Mucispirillum*	Increased during intestinal inflammation	[10]
Proteobaceria	*Helicobacter*	Induction of inflammation mainly by *H.pylori*	[11,12]

References for Supplementary information

1. Wexler, H.M. Bacteroides: the good, the bad, and the nitty-gritty. *Clinical microbiology reviews* **2007**, *20*, 593-621, doi:10.1128/CMR.00008-07.
2. Zhou, Y.; Zhi, F. Lower Level of<i> Bacteroides</i> in the Gut Microbiota Is Associated with Inflammatory Bowel Disease: A Meta-Analysis. *BioMed Research International* **2016**, *2016*, 5828959, doi:10.1155/2016/5828959.
3. Charlet, R.; Bortolus, C.; Sendid, B.; Jawhara, S. Bacteroides thetaiotaomicron and Lactobacillus johnsonii modulate intestinal inflammation and eliminate fungi via enzymatic hydrolysis of the fungal cell wall. *Scientific Reports* **2020**, *10*, 11510, doi:10.1038/s41598-020-68214-9.
4. Parker, B.J.; Wearsch, P.A.; Veloo, A.C.M.; Rodriguez-Palacios, A. The Genus Alistipes: Gut Bacteria With Emerging Implications to Inflammation, Cancer, and Mental Health. **2020**, *11*, doi:10.3389/fimmu.2020.00906.
5. Göker, M.; Gronow, S.; Zeytun, A.; Nolan, M.; Lucas, S.; Lapidus, A.; Hammon, N.; Deshpande, S.; Cheng, J.-F.; Pitluck, S., et al. Complete genome sequence of Odoribacter splanchnicus type strain (1651/6). *Stand Genomic Sci* **2011**, *4*, 200-209, doi:10.4056/sigs.1714269.
6. Stadlbauer, V.; Engertsberger, L.; Komarova, I.; Feldbacher, N.; Leber, B.; Pichler, G.; Fink, N.; Scarpatetti, M.; Schippinger, W.; Schmidt, R., et al. Dysbiosis, gut barrier dysfunction and inflammation in dementia: a pilot study. *BMC Geriatrics* **2020**, *20*, 248, doi:10.1186/s12877-020-01644-2.
7. Hu, S.; Wang, J.; Xu, Y.; Yang, H.; Wang, J.; Xue, C.; Yan, X.; Su, L. Anti-inflammation effects of fucosylated chondroitin sulphate from Acaudina molpadioides by altering gut microbiota in obese mice. *Food & function* **2019**, *10*, 1736-1746, doi:10.1039/C8FO02364F.
8. Cherbuy, C.; Bellet, D.; Robert, V.; Mayeur, C.; Schwiertz, A.; Langella, P. Modulation of the Caecal Gut Microbiota of Mice by Dietary Supplement Containing Resistant Starch: Impact Is Donor-Dependent. *Frontiers in microbiology* **2019**, *10*, 1234-1234, doi:10.3389/fmicb.2019.01234.
9. Yamamura, R.; Nakamura, K.; Kitada, N.; Aizawa, T.; Shimizu, Y.; Nakamura, K.; Ayabe, T.; Kimura, T.; Tamakoshi, A. Associations of gut microbiota, dietary intake, and serum short-chain fatty acids with fecal short-chain fatty acids. *Biosci Microbiota Food Health* **2020**, *39*, 11-17, doi:10.12938/bmfh.19-010.

10. Berry, D.; Kuzyk, O.; Rauch, I.; Heider, S.; Schwab, C.; Hainzl, E.; Decker, T.; Müller, M.; Strobl, B.; Schleper, C., et al. Intestinal Microbiota Signatures Associated with Inflammation History in Mice Experiencing Recurring Colitis. *Frontiers in microbiology* **2015**, *6*, 1408, doi:10.3389/fmicb.2015.01408.
11. Malfertheiner, P.; Link, A.; Selgrad, M. Helicobacter pylori: perspectives and time trends. *Nature Reviews Gastroenterology & Hepatology* **2014**, *11*, 628-638, doi:10.1038/nrgastro.2014.99.
12. Ménard, A.; Smet, A. Review: Other Helicobacter species. *Helicobacter* **2019**, *24 Suppl 1*, e12645, doi:10.1111/hel.12645.
